# Heterologous Reconstitution of Omega-3 Polyunsaturated Fatty Acids in *Arabidopsis*

**DOI:** 10.1155/2015/768478

**Published:** 2015-08-03

**Authors:** Sun Hee Kim, Kyung Hee Roh, Jong-Sug Park, Kwang-Soo Kim, Hyun Uk Kim, Kyeong-Ryeol Lee, Han-Chul Kang, Jong-Bum Kim

**Affiliations:** ^1^National Academy of Agricultural Science, Rural Development Administration, 370 Nongsaengnyeong-ro, Wansan-gu, Jeonju-si, Jeollabuk-do 560-500, Republic of Korea; ^2^National Institute of Crop Science, Rural Development Administration, Seodun-dong, Suwon 441-707, Republic of Korea

## Abstract

Reconstitution of nonnative, very-long-chain polyunsaturated fatty acid (VLC-PUFA) biosynthetic pathways in *Arabidopsis thaliana* was undertaken. The introduction of three primary biosynthetic activities to cells requires the stable coexpression of multiple proteins within the same cell. Herein, we report that C_22_ VLC-PUFAs were synthesized from C_18_ precursors by reactions catalyzed by Δ^6^-desaturase, an ELOVL5-like enzyme involved in VLC-PUFA elongation, and Δ^5^-desaturase. Coexpression of the corresponding genes (*McD6DES, AsELOVL5*, and *PtD5DES*) under the control of the seed-specific vicilin promoter resulted in production of docosapentaenoic acid (22:5 n-3) and docosatetraenoic acid (22:4 n-6) as well as eicosapentaenoic acid (20:5 n-3) and arachidonic acid (20:4 n-6) in *Arabidopsis* seeds. The contributions of the transgenic enzymes and endogenous fatty acid metabolism were determined. Specifically, the reasonable synthesis of omega-3 stearidonic acid (18:4 n-3) could be a useful tool to obtain a sustainable system for the production of omega-3 fatty acids in seeds of a transgenic T3 line 63-1. The results indicated that coexpression of the three proteins was stable. Therefore, this study suggests that metabolic engineering of oilseed crops to produce VLC-PUFAs is feasible.

## 1. Introduction

In both plants and animals, polyunsaturated fatty acids (PUFAs) are important membrane components that serve as universal cellular regulators, playing key roles in many cellular events. Very-long-chain (VLC) PUFAs are ≥20 carbons long (C_20_–C_22_) and have three or more methylene-interrupted* cis* double bonds in omega-3 (*ω*-3 or n-3) or omega-6 (*ω*-6 or n-6) arrangements (Table 1). Typically, VLC-PUFAs such as eicosapentaenoic acid (EPA; 20:5 n-3), arachidonic acid (ARA; 20:4 n-6), and docosahexaenoic acid (DHA; 22:6 n-3) are important nutritionally and as components of membrane phospholipids in specific tissues or as precursors for the synthesis of different groups of eicosanoid effectors. VLC-PUFAs are not only required for the development of the fetal nervous system but also contribute via a multiplicity of roles to the maintenance of health with increasing development and age, particularly by reducing the incidence of cardiovascular diseases [1, 2]. These fatty acids are either directly available as components of the diet or produced from the two essential fatty acids: *α*-linolenic acid (ALA; 18:3 n-3) and linoleic acid (LA; 18:2 n-6). Since mammalians and human cells are unable to synthesize ALA and LA from precursors, they are defined as essential PUFA. In humans, conversion of ALA and LA into their metabolites is extremely low and several factors, including age, gender, immune state, alcohol, and smoking, may influence LA and ALA metabolism. Thus, the major source of VLC-PUFAs is diet. Reliable dietary sources of VLC-PUFAs are fish oils, whereas ALA and LA are found predominantly in green vegetables and some plant oils, which do not contain VLC-PUFAs. Fish stocks are decreasing throughout the world, raising questions regarding the sustainability of this source of VLC-PUFAs, and this has resulted in strong interest in the production of long-chain PUFAs in plants [3]. This goal may be realized by the introduction of VLC-PUFA biosynthetic pathways into annual oilseed crops.

**Figure 1:**
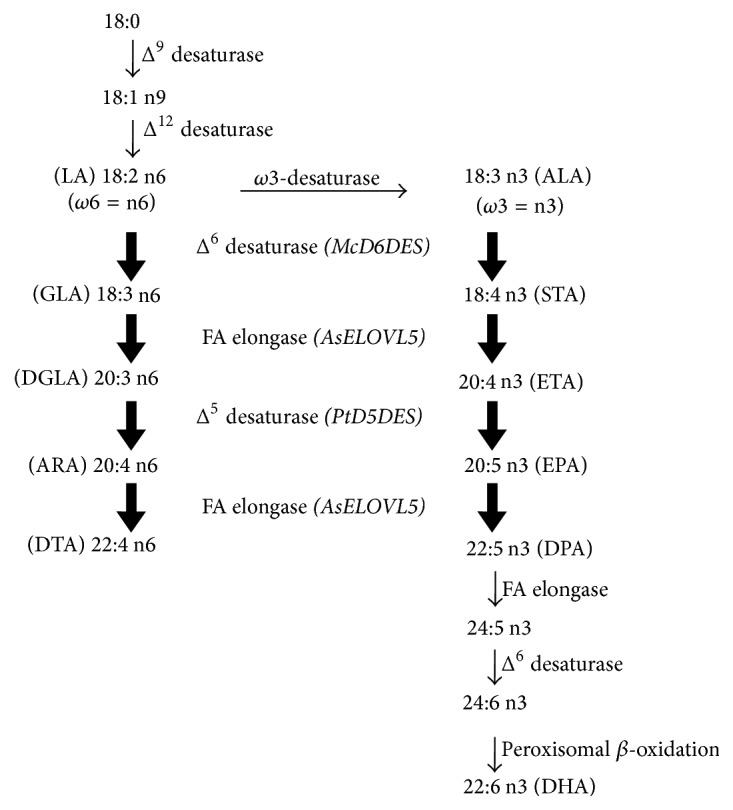
Metabolism of the two series of polyunsaturated fatty acids (PUFAs). Eicosapentaenoic acid (EPA, 20:5 n-3) is the main n-3 VLC-PUFA derived from the essential precursor *α*-linolenic acid (ALA, 18:3 n-3), and arachidonic acid (ARA, 20:4 n-6) is the main n-6 VLC-PUFA derived from the essential precursor linoleic acid (LA, 18:2 n-6). VLC-PUFAs are synthesized by successive elongations and desaturations. The key enzymes involved in this work are indicated in the pathway with large bold arrows.

The conversion of C_18_ PUFAs into EPA or ARA requires three consecutive enzymatic steps: Δ^6^-desaturation, PUFA elongation, and Δ^5^-desaturation (Figure 1). ALA and LA are desaturated by Δ^6^-desaturase to form stearidonic acid (STA; 18:4 n-3) and *γ*-linolenic acid (GLA; 18:3 n-6), respectively [4–7]. Next, STA and GLA are converted to eicosatetraenoic acid (ETA; 20:4 n-3) and dihomo-*γ*-linolenic acid (DGLA; 20:3 n-6), respectively, by an ELOVL5-like fatty acid elongase [8–11]. Finally, Δ^5^-desaturase converts ETA and DGLA into EPA and ARA, respectively [12–14]. Additionally, VLC-PUFAs such as EPA and ARA are further converted to docosapentaenoic acid (DPA; 22:5 n-3) and docosatetraenoic acid (DTA; 22:4 n-6) by ELOVL5 [10, 11, 15–19] or an ELOVL2-like fatty acid elongase [20] and then desaturated by Δ^4^-desaturase to give DPA and DHA [21, 22].

Indeed over recent years researchers have endeavored to reconstruct the VLC-PUFA biosynthetic pathways in plants [23–28]. In our previous study, we reconstituted the EPA and ARA biosynthetic pathways in yeast [11]. Here, we report the production of C_22_ VLC-PUFAs as well as EPA and ARA in seeds of transgenic* Arabidopsis* plants expressing appropriate heterologous enzymes under the control of seed-specific promoters. Our study reports a new gene set required to direct the efficient synthesis of these fatty acids in transgenic seeds.

## 2. Materials and Methods

### 2.1. Plant Material and Growth Conditions

Wild-type and transgenic plants were raised simultaneously from seeds in pots with soil.* Arabidopsis thaliana*, Columbia (Col-0) ecotype, was grown in a controlled environment chamber under the following conditions: 22°C day/18°C night, 70% humidity, and a 16 h photoperiod (250 *µ*mol m^−2^ s^−1^).

### 2.2. Construction of the Expression Vector

To produce VLC-PUFAs from ALA and LA through the n-3 and n-6 pathways, we needed a Δ^6^-desaturase, a fatty acid elongase, and a Δ^5^-desaturase. We identified genes encoding these enzymes in a variety of VLC-PUFA-producing organisms such as fish and algae in previous research [6, 7, 10]. We engineered a series of plant transformation constructs containing a Δ^6^-desaturase gene (*D6DES*) from the pike eel,* Muraenesox cinereus* [7]; a polyunsaturated fatty acid elongase gene (*ELOVL5*) from blackhead seabream,* Acanthopagrus schlegelii* [10]; and a Δ^5^-desaturase gene (*D5DES*) from a microalga,* Phaeodactylum tricornutum* strain KMCC B-128 (GenBank accession number GQ352540), all under the control of seed-specific promoters. The vector pGEM7Zf was used for construction of intermediate expression vectors. The primer sequences and other PCR-related information are summarized in Table 2.

**Figure 2:**
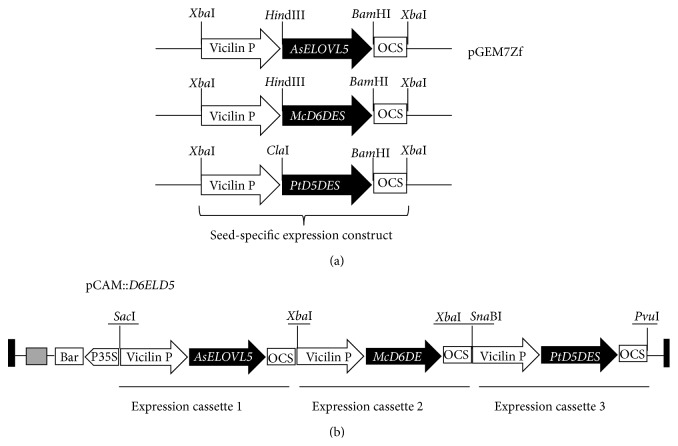
Schematic of constructs used for coexpression of the genes* McD6DES*,* AsELOVL5*, and* PtD5DES* in seeds of* Arabidopsis*. (a) The intermediate vector pGEM7Zf, used for individual seed-specific expression constructs. (b) The seed-specific expression vector* D6ELD5*, designed for easy coexpression of multiple genes. Vicilin P: vicilin promoter; OCS:* octopine synthase* terminator; bar, used to confer Basta resistance to plants under the control of a CaMV 35S promoter (P35S).

For the construction of expression vectors containing* McD6DES*,* AsELOVL5*, or* PtD5DES* under a seed-specific vicilin promoter, an expression construct containing a vicilin promoter, multiple cloning site, and* octopine synthase* (*OCS*) terminator from a vicilin cloning cassette was incorporated into pGEM7Zf. The individual* McD6DES*,* AsELOVL5*, and* PtD5DES* coding sequences were inserted into the* Hin*dIII and* Bam*HI or* Cla*I and* Bam*HI sites of the modified pGEM7Zf vector (Figure 2(a); Table 2 for primer set II). The fragment containing the vicilin promoter,* McD6DES*, and* OCS* terminator was isolated from the modified pGEM7Zf vector using a unique* Xba*I site and then ligated into the plant transformation vector pCAMBIA3300 (Cambia, Canberra, Australia) to form the seed-specific expression vector pCAM::*McD6DES*.

For the easy coexpression of the three genes in a single transformation step, one recombinant plasmid containing all three expression cassettes was constructed using an In-Fusion Advantage PCR Cloning Kit (Clontech, Mountain View, USA) according to the manufacturer's instructions. The vector was linearized with restriction enzymes. To amplify a target gene expression cassette, the 5′ end of the primer was designed to contain a 15 bp sequence homologous to the sequence at one end of the linearized vector. The 3′ end of the primer contained a sequence specific to the target gene (see primer set III in Table 2). The PCR product P_vic_AsELOVL5OCS was subcloned into pCAM::*McD6DES* linearized at the unique* Sac*I site to yield the plasmid pCAM::*McD6DES*–*AsELOVL5*. Finally, the recombinant plasmid pCAM::*D6ELD5* was generated by inserting the P_vic_PtD5DESOCS fragment into pCAM::*McD6DES*–*AsELOVL5* linearized at the unique* Pvu*I site introduced into P_vic_AsELOVL5OCS (Figure 2(b)).

### 2.3. Plant Transformation

The engineered expression construct was transformed into* Agrobacterium tumefaciens* strain GV3101 [29] by the freeze-thaw method and cultured at 28°C on a rotary shaker to the appropriate growth phase.* A. thaliana* ecotype Columbia was transformed with the pCAM::*D6ELD5* construct by the floral dipping method [30, 31]. Briefly, freshly opened flower buds were dipped in* A. tumefaciens* solution for 15 s, wrapped in plastic film, and left overnight in the dark at 22°C, after which the plastic was removed.

### 2.4. Generation of Transgenic Plants

Transgenic* Arabidopsis* lines were generated by 0.3% Basta (glufosinate) selection. Selection of transgenics was performed by spraying seedlings with 0.3% Basta upon emergence and twice afterwards at 3-day intervals. Transgenic plants displayed tolerance to Basta, whereas the untransformed control plants were severely damaged and died following Basta treatment. Basta-resistant plants were transferred to pots and grown to maturity. Plants were observed during growth for the presence of visible phenotypes. Copy number of the T-DNA insertion was not determined. In all cases, no phenotypic alterations of the plants were observed upon modification of the seed oil composition.

### 2.5. Verification of pCAM::*D6ELD5* in Transgenic* Arabidopsis*

To confirm the presence of all three transgenes in transgenic* Arabidopsis* plants, we performed PCR using primer set I (see Table 2). PCR was conducted on genomic DNA from young transgenic leaves using* AccuPower* Multiplex PCR PreMix (Bioneer Corp., Daejeon, Korea). The primers of primer set I were mixed within a single tube for DNA amplification and produced three different bands for high-throughput screening (data not shown).

Quantitative RT-PCR (qRT-PCR) was used to measure the coexpression of the three genes. At 9-10 days after flowering, siliques were taken from nine transgenic* Arabidopsis* plants differing in LC-PUFA output, and total RNA was extracted according to the method of Oñate-Sánchez and Vicente-Carbajosa [32]. Total RNA (1 *μ*g) was reverse-transcribed into cDNA using the PrimeScript RT Reagent Kit with gDNA Eraser (Takara Bio, Shiga, Japan). The expression of the three genes was investigated with qRT-PCR using the specific primers shown in Table 2. The PCR product size was 150 bp for all three genes. The samples were amplified using SYBR* Premix Ex Taq* II (Takara) under the following conditions: 30 s of initial denaturation at 95°C and 40 cycles of 5 s at 95°C and 34 s at 60°C. All reactions were performed in triplicate. The relative amounts of mRNA were calculated using the comparative C_T_ method (User Bulletin number 2; Applied Biosystems, Foster City, CA, USA). The mRNA levels were normalized to that of* ACTIN1*, a housekeeping gene. Thermal cycling and fluorescence detection were performed with a 7300 Real-Time PCR System (Applied Biosystems).

### 2.6. Fatty Acid Analysis

One hundred milligrams of seeds were homogenized using a mortar and pestle with 5 mL of MeOH/CHCl_3_ (2 : 1, v/v) and 1 mg PDA (pentadecanoic acid in MeOH) as an internal standard. Fatty acid methyl esters (FAMEs) were prepared with a lipid extraction method and analyzed by gas chromatography (GC) according to our previous study [6]. The FAMEs were identified by reference to peaks of well-characterized commercial standards (Supelco; Sigma-Aldrich, Pennsylvania, USA), based on the GC retention time, and quantified using computer software. All analyses were performed in triplicate and replicated three times. Methanolic base (Sigma-Aldrich Canada Ltd., Ontario, Canada) and FAME standards (PUFA number II, 47015-U; PUFA number III, 47085-U; Supelco) were used as the reference standards.

## 3. Results and Discussion

### 3.1. Establishment of the Recombinant Plasmid pCAM::*D6ELD5* in* Arabidopsis*

To optimize the production of VLC-PUFAs in oilseed plants, we conducted a stepwise engineering approach to generate a range of transgenic* Arabidopsis* lines carrying three genes, with each gene under the control of a seed-specific promoter. The expression construct (designated pCAM::*D6ELD5*) contained the minimal set of genes required for the synthesis of n-3 and n-6 C_22_ VLC-PUFAs (e.g., DPA and DTA) from endogenous C_18_ substrates (represented schematically in Figures 1 and 2(b)). The construct was verified by restriction analysis and sequencing of the resultant clones and introduced into* Arabidopsis* plants via floral dip transformation. From the T1 plants, 160 independent transgenic lines were selected with Basta. PCR analysis of genomic DNA revealed the presence of all three genes (*McD6DES*,* AsELOVL5*, and* PtD5DES*) in 104 of the selected plants (data not shown). Mature seeds from Basta-selected T2 plants were analyzed by GC for total fatty acid composition. No attempt was made to isolate homozygous lines from subsequent Basta-selected progeny.

### 3.2. Functional Analysis of PCAM::*D6ELD5* in* Arabidopsis*

We examined the effect of this construct on functionality by examining the fatty acid composition in transgenic seeds. To reconstitute the biosynthetic pathway of C_22_ VLC-PUFAs from C_18_ precursors, the* McD6DES*,* AsELOVL5*, and* PtD5DES* open reading frames (ORFs) were coexpressed in* Arabidopsis* (Figure 2(b)). The transgenic seeds coexpressing the three ORFs produced DPA and DTA, in addition to STA, ETA, and EPA of n-3 PUFAs and GLA, DGLA, and ARA of and n-6 PUFAs (Figure 1 and Table 3). These nonnative PUFAs were not detected in wild-type* Arabidopsis* seeds. These results demonstrate that the coexpression of* M. cinereus* Δ^6^-desaturase (McD6DES), an ELOVL5-like enzyme involved in VLC-PUFA elongation (AsELOVL5), and Δ^5^-desaturase (PtD5DES) successfully reconstituted the n-3 and n-6 pathways in a heterologous system. Unexpectedly, however, our product of LC-PUFA was detected with only low levels in seeds (Table 3). As seen in Table 4, these experiments demonstrated the viability of using transgenic manners to modify seed oil PUFA content. Abbadi et al. [23] showed the analysis of transgenic seeds for tobacco and linseed. They described that different substrate requirements, namely, phospholipid-linked substrates for desaturases and acyl-CoA for elongases, resulted in a rate-limiting flux through the alternating desaturation and elongation steps. In addition, Kinney et al. [24] demonstrated that use of the endogenous acyltransferases which could accept nonnative substrates produced high level EPA in transgenic soybean (*Glycine max*). Cheng et al. [25] investigated the effects of host species on EPA biosynthesis using* Brassica carinata*. Moreover, Robert et al. [26] used the way to target both desaturase and elongase activities in one pool for bypassing the acyl exchange bottleneck. Hoffmann et al. [27] also tried to avoid the acyl exchange bottleneck by using acyl-CoA-dependent desaturases. However, disappointingly the seed levels of target VLC-PUFAs were low like our seeds. Our* M. cinereus* Δ^6^-desaturase displayed preference for the n-3 substrate ALA, acting on this substrate with an efficiency of 21.3% compared to the n-6 substrate LA, of which only 7.0% was converted (Table 4). The* A. schlegelii* VLC-PUFA elongase demonstrated that this gene product has highly efficient activities (C18-elo and C20-elo). Yet although 42.6% of STA was elongated, only 6.5% of ETA was converted to EPA. It could be a likely explanation that repeat identical cassettes in the same orientation tend to be unstable, especially for the third cassette onward (D5-des in this case; Figure 2(b)). In addition, another approach is to use a strong acyl-CoA-dependent Δ^5^-desaturase. We will be interested in seeing the capacity to identify another optimal combination of FA biosynthetic activities for the production of VLC-PUFAs in plants using methods of Petrie et al. [28].

### 3.3. Selection of Transgenic* Arabidopsis*

The 160 independent lines from the first Basta selection were grouped together in 65 groups of two or three lines. Analysis of total FAMEs from the seeds of T2 lines in 28 of the 65 groups indicated that plants expressing the pCAM::*D6ELD5* construct accumulated nonnative VLC-PUFAs (n-3 PUFAs: STA, ETA, EPA, and DPA; n-6 PUFAs: GLA, DGLA, ARA, and DTA) (Table 3). In lines of groups 12, 18, 33, 63, and 64 (named EPA group lines), EPA represented about 0.2% of total fatty acids; this level was higher than that observed in other* Arabidopsis* T2 lines. EPA accounted for 0.1% of total fatty acids in transgenic lines of groups 11, 14, 15, 20, 21, 23, 27, 41, 44, and 65. No EPA or only trace levels of EPA were detected in transgenic lines of groups 1, 2, 5, 6, 24, 26, 28, 39, 43, 45, 57, 61, and 62. The levels of ARA (n-6) were similar to those of EPA (n-3). Other nonnative PUFAs of STA, ETA, and DPA were shown by 4%, 3%, and 0.4% of total fatty acids in EPA group lines, respectively. Otherwise GLA, DGLA, and DTA were about 2%, 3%, and 0.1% of total fatty acids, respectively. These fatty acids are active in substrate of EPA or ARA products in metabolism of VLC-PUFAs (but not DPA and DTA) (Figure 1). The EPA group lines preferentially produced omega-3 (n-3) PUFAs rather than omega-6 (n-6). These results were to investigate the effect of introduction of the* McD6DES* gene which could be a useful tool for omega-3 synthesis. Our previous study reported that the* McD6DES* gene was a sustainable system for the production of dietary omega-3 fatty acids [7]. For subsequent experiments, we selected transgenic lines of groups 33, 44, 63, and 64, which produced some DPA and DTA in addition to EPA and ARA.

### 3.4. Quantification of Fatty Acids

Accumulation of nonnative fatty acids was monitored in the T3 generation derived from the selected T2 plants (Table 5). Seeds of the transgenic T3 line 63-1 had the highest level of EPA, at 0.4% of total seed fatty acids; this represented a twofold increase in EPA level compared with seeds of the transgenic T2 line (Tables 3 and 5). The level of ARA in seeds of line 63-1 was 0.3% of total fatty acids, which was similar to the level found in T2 seeds. Levels of EPA and ARA in transgenic lines 33-1, 33-2, 33-4, 63-5, and 64-3 were half of those in line 63-1, in the range of 0.1–0.3% of total fatty acids (Table 5). Transgenic lines 44-1, 44-4, and 44-5 had only minor levels of EPA and ARA, in addition to DPA and DTA. In addition to having a higher level of EPA, seeds of line 63-1 also displayed increased levels of other LC-PUFAs, suggesting variation in the endogenous channeling of fatty acids into either pathway.

STA and GLA are intermediates in the early stages of EPA and ARA synthesis [33, 34], respectively, in the metabolism of VLC-PUFAs (Figure 1). STA in seeds of line 63-1, at 5.6% of total seed fatty acids, represented one and a half times increase compared with seeds of the transgenic T2 line (Tables 3 and 5). The level of STA was a threefold increase to GLA of line 63-1 which was similar to the level found in T2 seeds. ETA and DGLA were detected with similar level of total lipids in T3 plant of line 63-1, but ETA was increased by one times more compared with T2 plant. These contents are important for the production of EPA by the action of Δ^5^-desaturase [35–37]. Use of* P. tricornutum* Δ^5^-desaturase reached with 9.5% and 8.6% to EPA and ARA from substrates conversion of ETA and DGLA, respectively (Table 5).

DPA and DTA were 0.3 and 0.1% of the total fatty acids, respectively, in the transgenic T3 line 63-1. This suggests that DPA will be an important component of future metabolic engineering strategies for producing DHA with a combination of genes encoding Δ^4^-desaturase in plants [38]. These results showed that transgenic line 63-1 of T3 plants was more stable than T2 plants for expressing appropriate heterologous enzymes and also a sustainable system for the production of dietary omega-3 fatty acids. To study variation in the endogenous capacity to synthesize VLC-PUFAs, we analyzed the coordinated tissue-specific expression of multiple genes in seeds of transgenic T3 plants.

### 3.5. Heterologous Expression of pCAM::*D6ELD5* in* Arabidopsis*

**Figure 3:**
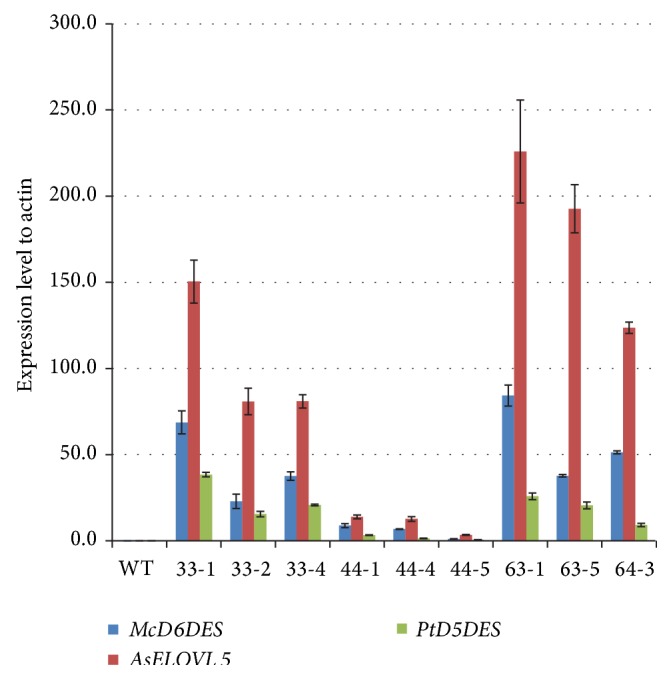
Analysis of pCAM::*D6ELD5* transcript accumulation in seeds of* Arabidopsis*. The genes* McD6DES*,* AsELOVL5*, and* PtD5DES* were assayed for transcript abundance. Data were normalized to* ACTIN1* mRNA levels and expressed as 2^−ΔΔCT^. Mean values obtained from three independent experiments are shown by the line.

After three rounds of Basta selection, nine independent transgenic T3 lines were obtained. The expression levels of the three transgenes in these lines were analyzed by qRT-PCR (Figure 3). The analysis indicated that the expression levels of the three genes encoded by* D6ELD5* were related to variation in the output of LC-PUFAs in transgenic seeds (Table 5 and Figure 3; see also the schematic representation in Figure 1). Overall levels of expression in line 63-1 were much higher than those in other transgenic lines, and the expression of* AsELOVL5* was highest in this line. The expression levels were quite different among the genes, with* AsELOVL5* being expressed at much higher levels than* McD6DES* or* PtD5DES*. The accumulation of ETA (or DGLA) and DPA (or DTA) was due to the activity of the elongase encoded by* AsELOVL5*. The sequential reactions catalyzed by the elongase, STA → ETA and EPA → DPA in the n-3 pathway and GLA → DGLA and ARA → DTA in the n-6 pathway, provide an abundance of precursor fatty acids for EPA and ARA synthesis (in the first reaction) and a progenitor of DHA (in the second reaction). Because the ELOVL5-like elongase yields both product and substrate, the high level of expression of* AsELOVL5* could provide some insight into the reasons for variation in the output of LC-PUFAs. Use of the efficient* A. schlegelii* VLC-PUFA elongase resulted in substrates conversion from STA and EPA to ETA and DPA reaching 40.4% and 42.9% in the n-3 pathway and from GLA and ARA to DGLA and DTA reaching 58.2% and 25.0% in the n-6 pathway, respectively (Table 5).

The step catalyzed by Δ^6^-desaturase is considered the rate-limiting step in the conversion of dietary ALA or LA to VLC-PUFAs [39, 40]. STA and GLA are produced with the desaturation of ALA and LA, respectively, by Δ^6^-desaturase (McD6DES).* McD6DES* was most highly expressed in line 63-1, and STA and GLA accumulated in this line. The STA accumulation was, as stated above, higher than GLA. These results were also shown in the conversion efficiency. Use of the efficient* M. cinereus* Δ^6^-desaturase of line 63-1 resulted in substrates conversion from ALA and LA to STA and GLA reached 26.8% and 7.7%, respectively (Table 5). However, the accumulation of EPA and ARA, which are products of ETA and DGLA desaturation, respectively, could not be directly related to the level of* PtD5DES* expression. Although increased levels of EPA and ARA were observed in line 63-1, the expression level of* PtD5DES* in line 63-1 was below that in line 33-1. It may be that a number of different factors regulate the accumulation of Δ^5^-desaturated fatty acids in the seeds. Therefore, the abundance of STA enabled abundant accumulation of EPA, and high expression of* AsELOVL5* resulted in the accumulation of EPA and DPA.

## 4. Conclusion

The modification of the lipid profile of oilseeds is an area of interest because the end-products have significant commercial value including foods, pharmaceuticals, or industrial raw material. However, the manipulation of plant seed oil composition is still a challenge. Because higher plants have no endogenous capacity for synthesis of VLC-PUFAs, we constructed a single recombinant plasmid from three gene expression cassettes to coexpress three genes using a single transformation step. The three primary enzymes needed for biosynthesis of VLC-PUFAs were expressed in transgenic* Arabidopsis* seeds as discrete transcription and translation products. The metabolism of endogenous cellular products, ALA and LA, to VLA-PUFAs was achieved in transgenic seeds by a series of desaturation and elongation reactions. Particularly, the introduction of the* McD6DES* gene could be a useful tool for omega-3 (n-3) STA synthesis is thought to be a rate-limiting step. Therefore, this study demonstrates that the success of this required expression of multiple genes, as three sequential nonnative enzymatic reactions are involved in the conversion of native plant FAs to VLC-PUFAs.

## Figures and Tables

**Table 1:** Abbreviations of PUFAs nomenclature used in this study.

n-3 PUFA	n-6 PUFA
ALA (*α*-linolenic acid; C_18:3_ Δ^9,12,15^)	LA (linoleic acid; C_18:2_ Δ^9,12^)
STA (stearidonic acid; C_18:4_ Δ^6,9,12,15^)	GLA (*γ*-linolenic acid; C_18:3_ Δ^6,9,12^)
ETA (eicosatetraenoic acid; C_20:4_ Δ^8,11,14,17^)	DGLA (dihomo-*γ*-linolenic acid; C_20:3_ Δ^8,11,14^)
EPA (eicosapentaenoic acid; C_20:5_ Δ^5,8,11,14,17^)	ARA (arachidonic acid; C_20:4_ Δ^5,8,11,14^)
DPA (docosapentaenoic acid; C_22:5_ Δ^7,10,13,16,19^)	DTA (docosatetraenoic acid; C_22:4_ Δ^7,10,13,16^)

**Table 2:** Primers used for this study. The data include sequences and annealing temperatures (*T*_*a*_) for the primer pairs.

Primer set	Primer name	Sequence (5′ → 3′)	*T*_*a*_
I	McD6D-106F	TGG TTG GTA ATC GAC AGA AAG GTG T	65°C
	McD6D-730R	TCT TTC CTA GCA CAA AGG TGT GGA G	
	AsELO-ORF-F	ATGGAGACCTTCAATCACAAACTGAACGTTTAC	
	AsELO-ORF-R	TCAATCCACTCTCAGTTTCTTGTGTGCAGTGT	
	PtD5D-ORF-F	ATGGCTCCGGATGCGGATAAGCTT	
	PtD5D-ORF-R	TTACGCCCGTCCGGTCAAGGGATTTT	

II	Hind-McD6D-F	CCC AAG CTT ATG GGA GGT GGA GGC CAG	62°C
	BamH-McD6D-R	CGG GAT CCT CAT TTA TGG AGA TAA GCA TCC	
	Hind-AsELO-F	CCC AAG CTT ATG GAG ACC TTC AAT CAC	
	BamH-AsELO-R	CGG GAT CCT CAA TCC ACT CTC AGT TT	
	Cla-PtD5D-F	CTT TAT TCA TCG ATA TGG CTC CGG ATG CGG AT	
	BamH-PtD5D-R	GCA GGA CTC TAG GAT CCT TAC GCC CGT CCG GTC AA	

III	Infu-AsELOVL5-F	GAT TAC GAA TTC GAG CTC GCG GCC GCT AAT ATT TTG CAA AAA	68°C
	Infu-AsELOVL5-R	CCC CGG GTA CCG AGC TCG TCC TGC TGA GCC TCG A	
	Infu-PtD5D-F	GCG AAG AGG CCC GCA CTA CGT ATA ATA TTT TGC AAA AAG A	
	Infu-PtD5D-R	CAA CTG TTG GGA AGG GCC AAT TGG TCC TGC TGA GCC TCG A	

qPCR	AtACT1 537F	TCT TGA TCT TGC TGG TCG TG	60°C
	AtACT1 707R	GAG CTG GTT TTG GCT GTC TC	
	McD6D 223F	TTC CAT CCA GAG CCT GAC TT	
	McD6D 371R	CCC TCC CTC TCT ACC TCC TC	
	AsELO 720F	CAC GCT AAT TTT CCT GTT CTC A	
	AsELO 871R	GTT TCT TGT GTG CAG TGT GCT	
	PtD5D 782F	GAT ACT GGT TGT CCG CTG TCT	
	PtD5D 935R	ATC ACG TTC ACC GCA ATG TA	

Restriction enzyme sites are underlined.

**Table 3:** Fatty acids composition (% w/w) produced by transgenic *Arabidopsis* T2 lines expressing pCAM::*D6ELD5* construct.

T2 group lines	Sample weight	16:0	18:0	18:1	LA	GLA	ALA	STA	20:1	DGLA	ARA	ETA	EPA	DTA	DPA
WT	0.1	8.5 ± 0.3	3.6 ± 0.2	15.1 ± 0.3	31.0 ± 0.3	0.0 ± 0.0	22.3 ± 0.1	0.0 ± 0.0	19.4 ± 0.3	0.0 ± 0.0	0.0 ± 0.0	0.0 ± 0.0	0.0 ± 0.0	0.0 ± 0.0	0.0 ± 0.0
1	0.1	8.6 ± 0.2	4.4 ± 0.1	17.1 ± 0.1	30.8 ± 0.1	0.6 ± 0.1	18.0 ± 0.1	1.3 ± 0.3	17.4 ± 0.3	0.7 ± 0.3	0.1 ± 0.0	1.0 ± 0.2	0.0 ± 0.0	0.0 ± 0.1	0.1 ± 0.0
2	0.1	8.4 ± 0.3	4.3 ± 0.2	16.6 ± 0.3	30.2 ± 0.4	0.7 ± 0.2	19.1 ± 0.4	1.4 ± 0.1	17.2 ± 0.1	0.9 ± 0.1	0.1 ± 0.1	1.1 ± 0.4	0.0 ± 0.0	0.0 ± 0.1	0.1 ± 0.1
5	0.1	8.8 ± 0.1	4.4 ± 0.3	15.6 ± 0.2	31.2 ± 0.1	0.7 ± 0.3	18.2 ± 0.4	1.5 ± 0.1	17.2 ± 0.1	1.0 ± 0.4	0.1 ± 0.1	1.2 ± 0.1	0.0 ± 0.0	0.0 ± 0.1	0.1 ± 0.0
6	0.1	8.6 ± 0.4	4.0 ± 0.3	15.9 ± 0.5	31.4 ± 0.2	0.2 ± 0.1	19.4 ± 0.2	0.4 ± 0.4	19.8 ± 0.3	0.1 ± 0.1	0.0 ± 0.0	0.2 ± 0.1	0.0 ± 0.0	0.0 ± 0.0	0.0 ± 0.0
11	0.1	9.5 ± 0.3	5.5 ± 0.1	14.1 ± 0.3	29.2 ± 0.1	1.6 ± 0.1	16.5 ± 0.3	3.6 ± 0.1	14.6 ± 0.4	2.3 ± 0.1	0.2 ± 0.1	2.5 ± 0.4	0.1 ± 0.0	0.0 ± 0.1	0.3 ± 0.0
12	0.1	9.8 ± 0.5	6.1 ± 0.2	14.6 ± 0.3	28.6 ± 0.2	1.9 ± 0.3	14.8 ± 0.3	4.1 ± 0.4	13.5 ± 0.3	2.8 ± 0.2	0.2 ± 0.0	3.1 ± 0.4	0.2 ± 0.0	0.0 ± 0.0	0.3 ± 0.0
14	0.1	9.2 ± 0.3	4.3 ± 0.4	14.7 ± 0.1	30.8 ± 0.1	1.0 ± 0.1	19.2 ± 0.1	2.4 ± 0.3	15.0 ± 0.1	1.4 ± 0.3	0.1 ± 0.1	1.9 ± 0.3	0.1 ± 0.0	0.0 ± 0.0	0.1 ± 0.0
15	0.1	8.8 ± 0.2	4.8 ± 0.3	15.5 ± 0.1	30.4 ± 0.1	1.1 ± 0.4	17.2 ± 0.2	2.9 ± 0.2	15.3 ± 0.1	1.6 ± 0.2	0.2 ± 0.1	2.0 ± 0.5	0.1 ± 0.0	0.0 ± 0.0	0.2 ± 0.0
18	0.1	10.1 ± 0.3	5.0 ± 0.2	12.3 ± 0.1	30.3 ± 0.2	1.8 ± 0.2	17.5 ± 0.4	3.6 ± 0.4	14.1 ± 0.1	2.2 ± 0.1	0.2 ± 0.2	2.4 ± 0.4	0.2 ± 0.0	0.0 ± 0.0	0.2 ± 0.0
20	0.1	9.4 ± 0.4	4.7 ± 0.4	13.0 ± 0.3	30.9 ± 0.4	1.2 ± 0.1	18.3 ± 0.2	2.5 ± 0.1	16.6 ± 0.3	1.4 ± 0.3	0.1 ± 0.1	1.6 ± 0.1	0.1 ± 0.0	0.0 ± 0.0	0.2 ± 0.0
21	0.1	8.8 ± 0.3	4.4 ± 0.1	16.6 ± 0.2	30.7 ± 0.4	1.1 ± 0.2	17.7 ± 0.4	2.2 ± 0.4	15.9 ± 0.4	1.0 ± 0.0	0.0 ± 0.0	1.4 ± 0.1	0.1 ± 0.0	0.0 ± 0.0	0.1 ± 0.0
23	0.1	8.7 ± 0.1	4.3 ± 0.5	15.0 ± 0.5	30.9 ± 0.1	0.5 ± 0.4	19.3 ± 0.1	1.2 ± 0.1	18.4 ± 0.3	0.7 ± 0.2	0.1 ± 0.0	0.8 ± 0.3	0.1 ± 0.0	0.0 ± 0.0	0.1 ± 0.0
24	0.1	8.8 ± 0.2	3.7 ± 0.4	14.2 ± 0.1	32.6 ± 0.3	0.1 ± 0.1	20.5 ± 0.1	0.2 ± 0.2	19.7 ± 0.1	0.1 ± 0.0	0.0 ± 0.0	0.1 ± 0.0	0.0 ± 0.0	0.0 ± 0.0	0.0 ± 0.0
26	0.1	8.7 ± 0.3	4.4 ± 0.3	15.4 ± 0.2	31.2 ± 0.2	0.6 ± 0.3	18.3 ± 0.4	0.9 ± 0.1	19.0 ± 0.2	0.8 ± 0.1	0.0 ± 0.0	0.7 ± 0.1	0.0 ± 0.0	0.0 ± 0.0	0.1 ± 0.0
27	0.1	8.6 ± 0.4	4.2 ± 0.3	14.2 ± 0.3	31.7 ± 0.1	0.8 ± 0.1	18.4 ± 0.4	1.5 ± 0.3	17.8 ± 0.4	1.2 ± 0.3	0.1 ± 0.0	1.4 ± 0.4	0.1 ± 0.1	0.0 ± 0.0	0.1 ± 0.0
28	0.1	8.7 ± 0.2	3.9 ± 0.2	14.5 ± 0.1	32.1 ± 0.1	0.2 ± 0.1	20.1 ± 0.2	0.4 ± 0.4	19.3 ± 0.4	0.3 ± 0.1	0.0 ± 0.0	0.4 ± 0.1	0.0 ± 0.0	0.0 ± 0.0	0.0 ± 0.0
33	0.1	9.0 ± 0.3	5.0 ± 0.5	15.1 ± 0.2	30.2 ± 0.2	2.1 ± 0.1	13.7 ± 0.1	3.4 ± 0.1	14.7 ± 0.2	3.2 ± 0.4	0.2 ± 0.0	3.0 ± 0.1	0.2 ± 0.0	0.0 ± 0.0	0.3 ± 0.1
39	0.1	8.5 ± 0.2	4.0 ± 0.3	15.9 ± 0.1	33.9 ± 0.1	0.1 ± 0.1	18.1 ± 0.2	0.2 ± 0.2	19.2 ± 0.1	0.0 ± 0.0	0.0 ± 0.0	0.1 ± 0.0	0.0 ± 0.0	0.0 ± 0.0	0.0 ± 0.0
41	0.1	9.1 ± 0.1	4.8 ± 0.2	13.4 ± 0.1	31.1 ± 0.3	1.5 ± 0.4	16.9 ± 0.3	2.8 ± 0.3	16.5 ± 0.4	1.7 ± 0.1	0.2 ± 0.1	1.8 ± 0.2	0.1 ± 0.1	0.0 ± 0.0	0.1 ± 0.0
43	0.1	8.9 ± 0.4	5.4 ± 0.1	15.1 ± 0.1	29.2 ± 0.1	1.9 ± 0.2	16.7 ± 0.2	3.7 ± 0.3	15.5 ± 0.1	1.8 ± 0.3	0.0 ± 0.0	2.0 ± 0.1	0.0 ± 0.0	0.0 ± 0.0	0.0 ± 0.0
44	0.1	9.1 ± 0.3	5.2 ± 0.3	13.2 ± 0.3	30.5 ± 0.2	1.5 ± 0.1	16.6 ± 0.3	2.6 ± 0.2	16.7 ± 0.1	2.1 ± 0.1	0.2 ± 0.0	2.0 ± 0.3	0.1 ± 0.0	0.1 ± 0.1	0.2 ± 0.1
45	0.1	9.0 ± 0.2	4.4 ± 0.2	15.4 ± 0.2	31.1 ± 0.1	0.9 ± 0.3	17.7 ± 0.3	1.8 ± 0.1	17.4 ± 0.2	1.2 ± 0.3	0.0 ± 0.0	1.1 ± 0.2	0.0 ± 0.0	0.0 ± 0.0	0.0 ± 0.0
57	0.1	9.2 ± 0.1	5.0 ± 0.3	13.6 ± 0.1	29.7 ± 0.1	1.1 ± 0.4	18.7 ± 0.4	2.6 ± 0.1	16.1 ± 0.4	1.7 ± 0.4	0.0 ± 0.0	2.2 ± 0.4	0.0 ± 0.0	0.0 ± 0.0	0.0 ± 0.0
61	0.1	9.1 ± 0.3	5.0 ± 0.4	14.2 ± 0.3	31.7 ± 0.5	1.2 ± 0.3	16.7 ± 0.3	2.7 ± 0.1	15.6 ± 0.1	1.9 ± 0.3	0.0 ± 0.0	2.1 ± 0.2	0.0 ± 0.0	0.0 ± 0.0	0.0 ± 0.0
62	0.1	11.2 ± 0.3	5.6 ± 0.3	0.0 ± 0.0	36.2 ± 0.1	1.9 ± 0.1	18.9 ± 0.3	4.3 ± 0.3	16.4 ± 0.3	2.6 ± 0.3	0.0 ± 0.0	2.9 ± 0.2	0.0 ± 0.0	0.0 ± 0.0	0.0 ± 0.0
63	0.1	9.5 ± 0.2	4.8 ± 0.2	14.0 ± 0.3	30.6 ± 0.2	2.3 ± 0.3	14.4 ± 0.3	3.9 ± 0.1	13.6 ± 0.4	3.1 ± 0.2	0.3 ± 0.0	2.9 ± 0.1	0.2 ± 0.0	0.1 ± 0.0	0.3 ± 0.0
64	0.1	9.3 ± 0.3	4.5 ± 0.3	14.3 ± 0.2	31.9 ± 0.1	2.1 ± 0.1	14.8 ± 0.1	3.0 ± 0.1	14.2 ± 0.4	2.8 ± 0.1	0.2 ± 0.1	2.4 ± 0.4	0.2 ± 0.0	0.1 ± 0.1	0.4 ± 0.0
65	0.1	8.7 ± 0.4	4.3 ± 0.4	16.8 ± 0.2	31.9 ± 0.2	1.3 ± 0.3	15.9 ± 0.2	2.6 ± 0.1	15.2 ± 0.3	1.8 ± 0.1	0.1 ± 0.0	1.1 ± 0.3	0.1 ± 0.0	0.0 ± 0.0	0.2 ± 0.0

Each value is the mean ± SD from three independent experiments. Total lipid is mg lipid g^−1^ dry cells.

**Table 4:** Comparison of published transgenic lines producing VLC-PUFAs and biosynthetic intermediates.

Reference	Plant species	Tissue	GLA	STA	DGLA	ARA	ETA	EPA	DPA
This work [63 transgenic T2 line in Table 3] (Conversion efficiencies)	*A. thaliana *	Seed	2.3 **(7.0%, D6-des)**	3.9 **(21.3%, D6-des)**	3.1 **(59.2%, C18-elo)**	0.3 **(8.8%, D5-des)**	2.9 **(42.6%, C18-elo)**	0.2 **(6.5%, D5-des)**	0.3 **(60%, C20-elo)**
Abbadi et al. [23]	*N. tabacum *	Seed	29.3	—	1.8	1.5	—	—	—
	*L. usitatissimum *	Seed	16.8	11.4	1.2	1.0	0.9	0.8	—
Kinney et al. [24]	*G. max *	Seed	11.7	1.1	10.1	2.2	2.4	19.6	0.8
Cheng et al. [25]	*B. carinata *	Seed	26.9	5.4	2.2	5.7	2.5	20.4	4.0
Robert et al. [26]	*A. thaliana *	Seed	0.6	1.8	1.9	1.6	0.4	3.2	0.1
Hoffmann et al. [27]	*A. thaliana *	Seed	>0.5	>0.1	0.8	0.1	0.9	0.05	—

Where shown, conversion efficiencies are calculated as [product]/[product + substrate] × 100. This value represents the overall percent of conversion of total substrate into total product formed.

**Table 5:** Fatty acids composition (% w/w) produced by transgenic *Arabidopsis* T3 lines expressing pCAM::*D6ELD5* construct.

Usual FA	WT	33-1	33-2	33-4	44-1	44-4	44-5	63-1	63-5	64-3

Fatty acid										
16:00	8.5 ± 0.3	8.8 ± 0.4	8.9 ± 0.1	8.7 ± 0.3	8.5 ± 0.2	8.6 ± 0.3	8.5 ± 0.1	9.4 ± 0.1	9.3 ± 0.3	9.4 ± 0.1
18:00	3.8 ± 0.1	4.2 ± 0.1	4.7 ± 0.1	4.8 ± 0.1	4.2 ± 0.2	4.2 ± 0.3	3.9 ± 0.4	5.3 ± 0.3	5.1 ± 0.2	5.1 ± 0.2
18:01	13.5 ± 0.1	12.6 ± 0.1	13.6 ± 0.1	13.3 ± 0.3	13.6 ± 0.1	13.4 ± 0.4	12.6 ± 0.3	13.5 ± 0.1	13.4 ± 0.3	13.4 ± 0.1
LA	29.6 ± 0.3	28.4 ± 0.1	27.5 ± 0.2	27.6 ± 0.4	30.5 ± 0.3	29.5 ± 0.2	29.8 ± 0.1	27.4 ± 0.3	28.0 ± 0.2	28.2 ± 0.4
ALA	22.9 ± 0.1	19.7 ± 0.4	19.6 ± 0.3	19.2 ± 0.1	20.9 ± 0.2	21.7 ± 0.3	22.9 ± 0.2	15.3 ± 0.1	17.5 ± 0.4	17.2 ± 0.3
20:01	21.7 ± 0.4	17.0 ± 0.1	16.8 ± 0.1	17.4 ± 0.1	20.9 ± 0.2	21.1 ± 0.2	21.6 ± 0.2	13.1 ± 0.3	17.0 ± 0.4	16.2 ± 0.1

Total	100	90.7	91.1	91	98.6	98.5	99.3	84	90.3	89.5

New *n*-3 PUFA										
STA	0.0 ± 0.0	3.3 ± 0.5	3.2 ± 0.4	3.1 ± 0.5	0.5 ± 0.1	0.6 ± 0.1	0.2 ± 0.0	5.6 ± 0.1 (26.8%, D6-des)	3.2 ± 0.1	3.5 ± 0.4
ETA	0.0 ± 0.0	2.2 ± 0.1	2.2 ± 0.1	2.2 ± 0.1	0.4 ± 0.1	0.4 ± 0.1	0.2 ± 0.0	3.8 ± 0.1 (40.4%, C18-elo)	2.3 ± 0.1	2.5 ± 0.3
EPA	0.0 ± 0.0	0.2 ± 0.2	0.2 ± 0.1	0.1 ± 0.0	0.0 ± 0.0	0.0 ± 0.0	0.0 ± 0.0	0.4 ± 0.0 (9.5%, D5-des)	0.2 ± 0.0	0.2 ± 0.0
DPA	0.0 ± 0.0	0.1 ± 0.0	0.1 ± 0.0	0.2 ± 0.0	0.0 ± 0.0	0.0 ± 0.0	0.0 ± 0.0	0.3 ± 0.0 (42.9%, C20-elo)	0.2 ± 0.0	0.2 ± 0.0

Total	0	5.8	5.7	5.6	0.9	1	0.4	10.1	5.9	6.4

New *n*-6 PUFA										
GLA	0.0 ± 0.0	1.3 ± 0.2	1.2 ± 0.1	1.3 ± 0.1	0.2 ± 0.1	0.2 ± 0.2	0.1 ± 0.1	2.3 ± 0.1 (7.7%, D6-des)	1.4 ± 0.3	1.7 ± 0.4
DGLA	0.0 ± 0.0	1.8 ± 0.4	1.7 ± 0.1	1.8 ± 0.2	0.3 ± 0.0	0.3 ± 0.0	0.2 ± 0.2	3.2 ± 0.5 (58.2%, C18-elo)	2.1 ± 0.4	2.2 ± 0.2
ARA	0.0 ± 0.0	0.3 ± 0.0	0.2 ± 0.0	0.2 ± 0.0	0.0 ± 0.0	0.0 ± 0.0	0.0 ± 0.0	0.3 ± 0.0 (8.6%, D5-dea)	0.2 ± 0.0	0.1 ± 0.0
DTA	0.0 ± 0.0	0.1 ± 0.0	0.1 ± 0.0	0.1 ± 0.0	0.0 ± 0.0	0.0 ± 0.0	0.0 ± 0.0	0.1 ± 0.0 (25.0%, C20-elo)	0.1 ± 0.0	0.1 ± 0.0

Total	0	3.5	3.2	3.4	0.5	0.5	0.3	5.9	3.8	4.1

Total new FA	0	9.3	8.9	9	1.4	1.5	0.7	16	9.7	10.5

Total FA	100	100	100	100	100	100	100	100	100	100

Each value is the mean ± SD from five independent experiments. Total lipid is mg lipid g^−1^ dry cells. Where shown, conversion efficiencies are calculated as [product]/[product + substrate] × 100. This value represents the overall percent of conversion of total substrate into total product formed.
